# Targeting the Symptom-Driving Level in Multilevel Lumbar Stenosis Using Unilateral Biportal Endoscopy: A Strategy Reappraisal

**DOI:** 10.3390/jcm15134875

**Published:** 2026-06-23

**Authors:** Insafe Mezjan, Aurore Sellier, François Lechanoine, Nacer Mansouri, Guillaume Lonjon, François-Xavier Ferracci, Louis-Marie Terrier, Philippe Cam, Anthony Melot, Joseph Cristini

**Affiliations:** 1 Department of Neurosurgery, Centre Hospitalier Régional Universitaire de Nancy, Université de Lorraine, 54000 Nancy, France; mezjan.insafe@outlook.com (I.M.); mansouri.nz@gmail.com (N.M.); 2 Department of Neurosurgery, Toulon Hyeres Saint Jean Private Hospital, 83100 Toulon, France; aurore.sellier@neurochirurgie.fr; 3 Department of Neurosurgery, Monticelli Private Hospital, Ramsay Santé, 13008 Marseille, France; fx.ferracci@gmail.com (F.-X.F.); louis.terrier@neurochirurgie.fr (L.-M.T.); drphilippecam@gmail.com (P.C.); anthony.melot@neurochirurgie.fr (A.M.); 4 NeuroNEC Brain & Spine, Strada di Paderna 2, 47895 Domagnano, San Marino; f.lechanoine@neuronec.org; 5 Department of Orthopedic Surgery, Orthosud, Clinique St-Jean Sud De France, Santecite Group, 34430 Montpellier, France; guilonjon@gmail.com; 6 Department of Neurosurgery, Clairval Private Hospital, Ramsay Santé, 13009 Marseille, France

**Keywords:** unilateral biportal endoscopy (UBE), multilevel lumbar spinal stenosis, minimally invasive spine surgery (MIS)

## Abstract

**Background/Objectives:** Multilevel lumbar spinal stenosis (MLSS) is frequently encountered in patients undergoing surgery for lumbar spinal stenosis, yet the optimal extent of decompression remains debated. While multilevel decompression (MLD) may address all radiological stenotic levels, it may also increase surgical invasiveness and operative time. Minimally invasive endoscopic techniques such as unilateral biportal endoscopy (UBE) allow for targeted decompression and facilitate staged surgical strategies. The aim of this study was to evaluate the clinical outcomes of selective single-level decompression (SLD) using UBE in patients presenting with MLSS. **Methods:** This retrospective monocentric observational study included consecutive adult patients with MLSS who underwent decompression using UBE between December 2022 and July 2025. MLSS was defined as the presence of at least two lumbar levels with Schizas grade B or higher stenosis. Patients undergoing prior lumbar surgery or presenting with non-degenerative pathology were excluded. Patients underwent either SLD targeting the symptom-driving level or MLD, depending on the surgical strategy. Patient-reported outcomes included the Oswestry Disability Index (ODI), lumbar visual analog scale (LVAS), and radicular visual analog scale (RVAS). **Results:** Among 305 patients operated on for lumbar spinal stenosis, 83 (27%) presented with MLSS and were included in the study. Seventy-four patients (89%) underwent initial SLD and nine (11%) underwent MLD. Among patients treated with SLD, 9 (12%) required a second decompression during follow-up, whereas 65 patients (88%) achieved favorable outcomes without further surgery. Across the entire cohort, ODI, LVAS, and RVAS improved significantly after surgery. Operative time was significantly longer in the MLD group (122 ± 28.1 min vs. 58.1 ± 12.0 min; *p* < 0.001). These findings support the feasibility of a symptom-driven selective decompression strategy for MLSS using UBE. In our cohort, most patients experienced meaningful functional improvement after SLD without requiring additional surgery. Although a staged approach may necessitate secondary intervention in a minority of patients, selective decompression may help limit surgical extent in carefully selected patients while preserving favorable clinical outcomes. **Conclusions:** Selective SLD using UBE was associated with significant clinical improvement in most patients with MLSS while reducing operative time and surgical extent. A stepwise strategy targeting the dominant symptomatic level may represent a feasible minimally invasive approach for selected patients with MLSS. Prospective studies are needed to confirm these findings.

## 1. Introduction

Lumbar spinal stenosis affects approximately 103 million persons worldwide [1]. The number of surgical procedures performed for this condition continues to increase, especially in the elderly population, reflecting demographic aging and the rising expectations for postoperative functional recovery [2].

Multilevel lumbar spinal stenosis (MLSS) is common, occurring in up to 40% of patients with lumbar stenosis [3]. The indication to decompress multiple levels versus addressing only the index level remains debated. A recent five-year follow-up study showed no clinically relevant differences in outcomes between single-level decompression (SLD) and multi-level decompression (MLD) [4], and a prospective multicenter study found that SLD was associated with better functional scores [5]. However, a major limitation of selective SLD in open procedures is the concern for revision surgery. Reintervention after open decompression is associated with substantial epidural fibrosis, increased risk of dural tears [6], and higher perioperative morbidity [7], which may discourage a selective surgical strategy.

Minimally invasive spinal endoscopy has profoundly modified the surgical management of degenerative lumbar spine disease by substantially reducing approach-related morbidity, with less trauma and diminished blood loss, and by improving rehabilitation outcomes [8,9]. Importantly, endoscopic decompression is inherently level-specific, with one surgical approach required per treated level [10]. Because endoscopic reintervention uses a distinct and minimally invasive surgical corridor, postoperative fibrosis from a previous procedure does not represent the same technical obstacle as in open surgery. As a result, staged or selective decompression strategies become feasible, potentially allowing surgeons to target only the clinically dominant level while preserving the option of safe reoperation if symptoms persist or recur.

Therefore, the aim of this study was to evaluate the outcome of an SLD using unilateral biportal endoscopy (UBE) in patients presenting with MLSS.

## 2. Material and Methods

### 2.1. Study Design and Participants

**Figure 1:**
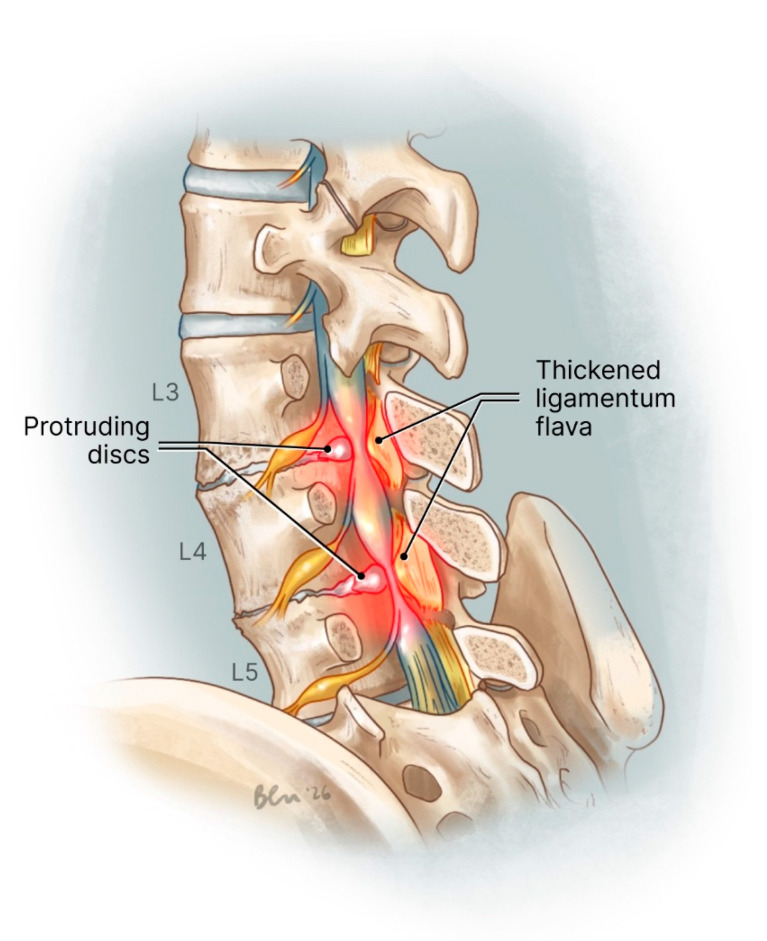
Schematic illustration of MLSS at the L3–L4 and L4–L5 levels, with thickened ligamentum flavum and disc protrusions contributing to the stenosis. Copyright of Bridget Xiao-Cha Lu, used with permission.

**Figure 2:**
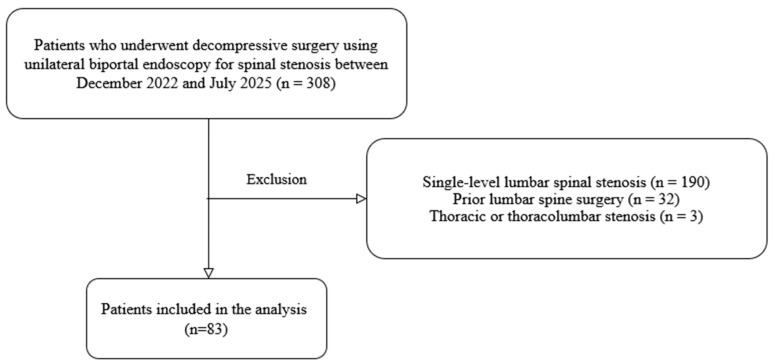
Flowchart.

This monocentric observational retrospective study included every consecutive adult patient who presented an MLSS (Figure 1) and underwent decompressive surgery using UBE between December 2022 and July 2025. MLSS was defined as the presence of two or more lumbar levels classified as Schizas grade B or higher [11]. Patients with previous lumbar surgery and non-degenerative pathology were excluded. Our flowchart is presented in Figure 2.

### 2.2. Surgical Technique

All patients were operated on under general anesthesia, in a prone position and guided by fluoroscopy. The surgical technique has previously been described in detail in Sellier et al. [10]. The final step of the decompression consisted of a foraminotomy with exposure of the ipsilateral and/or contralateral nerve roots when clinically relevant. In cases of associated disc herniation at the operated level, the herniated disc fragment was removed. All patients were operated on by the same experienced senior surgeon (J.C). The MLD group comprised patients who underwent a single surgical procedure with decompression of multiple stenotic levels, prior to implementation of the surgical strategy described below. The SLD group included patients who underwent targeted decompression of a single symptomatic level. Patients in the SLD group could undergo a second surgical procedure if further decompression was deemed necessary during follow-up.

**Figure 3:**
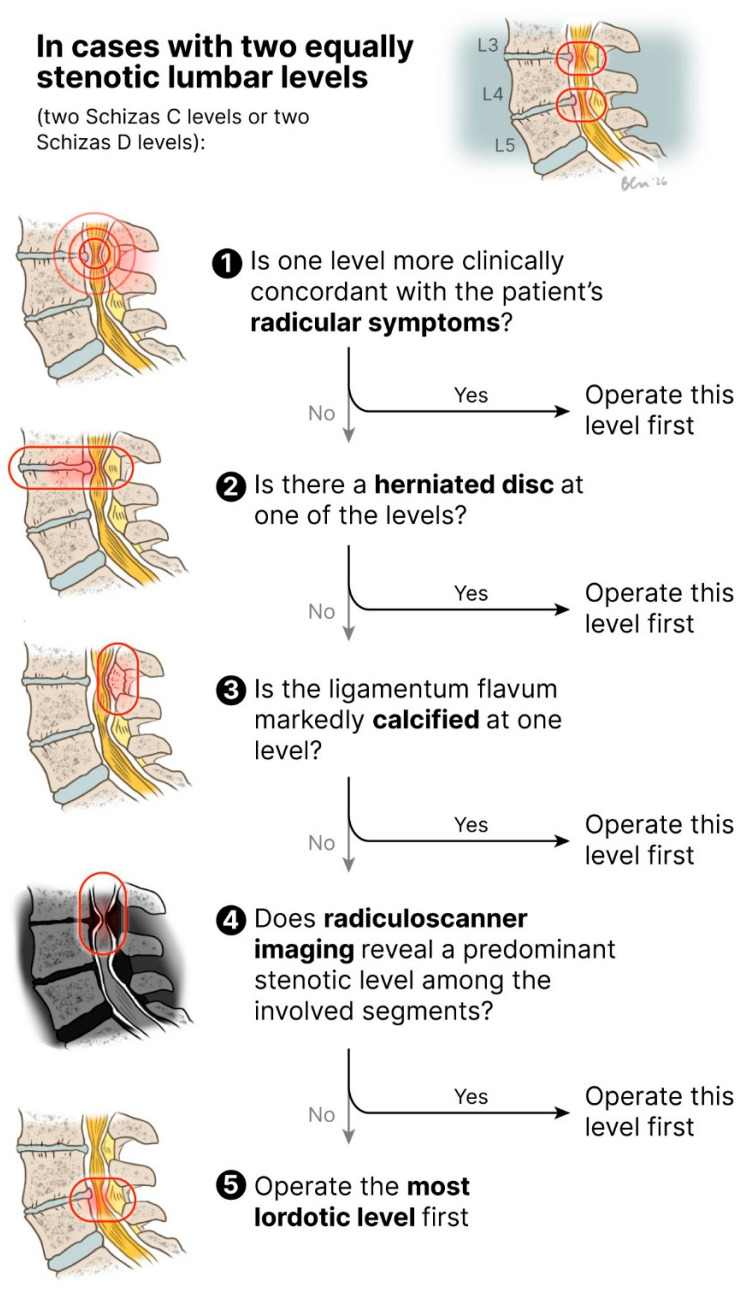
Decision-making algorithm to identify the symptom-driving level in case of MLSS. Copyright of Bridget Xiao-Cha Lu, used with permission.

**Figure 4:**
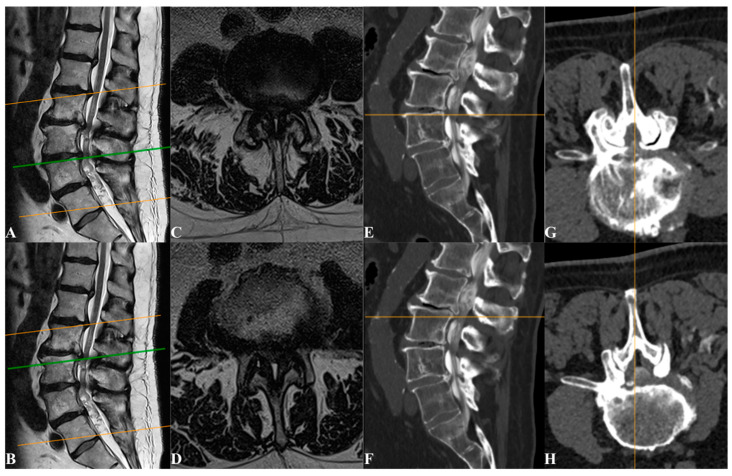
Clinical case illustration: A 73-year-old patient presenting with bilateral radicular leg pain and limited walking distance (<300 m) was diagnosed with MLSS at L2–L3 and L3–L4, graded Schizas C. All images represent preoperative imaging. (**A**,**B**) Sagittal T2-weighted MRI, with the green line indicating the level of the corresponding axial images. (**C**) Axial T2-weighted MRI at the L3–L4 level. (**D**) Axial T2-weighted MRI at the L2–L3 level. Patient’s symptoms, MRI, CT scanner imaging and clinical examination did not allow for identification of the symptom-driving level. (**E**,**F**) Sagittal radiculoscanner, with the orange line indicating the level of the corresponding axial images. (**G**) Axial radiculoscanner at the L3–L4 level. (**H**) Axial radiculoscanner at the L2–L3 level. The radiculoscanner demonstrated more severe stenosis at L3–L4 than at L2–L3. The patient therefore underwent decompression at L3–L4 and did not require additional surgery to date (2 years follow-up).

In the SLD group, the most severely stenotic level was targeted first. When multiple levels demonstrated equivalent severity of stenosis according to the Schizas classification [11], a predefined decision-making algorithm (Figure 3) was applied to identify and prioritize the symptom-driving level for initial decompression (Figure 4). The first step of the algorithm consisted of identifying whether one stenotic level demonstrated stronger clinico-radiological concordance with the patient’s symptoms than the other affected levels. Because central canal stenosis at two adjacent lumbar levels rarely allows for reliable clinical discrimination of the truly symptomatic level, particular attention was paid to the presence of associated foraminal stenosis when it involved the exiting nerve root at a level and side concordant with the patient’s dermatomal pain distribution. As surgery was never considered as a first-line treatment, all patients initially underwent conservative management, including medical treatment and image-guided infiltrations. Consequently, the response to selective nerve root blocks performed by interventional radiologists was also taken into account to help identify the symptomatic level and guide surgical decision-making. However, some interventional radiologists preferred facet joint infiltrations or sacrococcygeal hiatus epidural infiltrations in cases of severe stenosis, thereby limiting the diagnostic contribution of image-guided infiltrations for identifying the symptom-driving level. In cases where clinical findings did not allow for reliable identification of the operative level, the subsequent steps of the algorithm were applied.

### 2.3. Outcomes

The primary objective of this study was to evaluate the functional outcome of an SLD in patients presenting with MLSS. Secondary objectives were to assess the need for subsequent surgical intervention during follow-up.

### 2.4. Data Collection

Data were prospectively recorded using the medical software Follow Health (https://www.follow.fr/ (accessed on 3 June 2026), Version 3). Data collected for each patient included age at surgery, retirement/employment status, American Society of Anesthesiologists (ASA) score, history of spine surgery, clinical presentation, and radiological findings (number of stenotic levels and stenosis grade according to the Schizas classification [11]). Surgical variables included operative duration and perioperative and postoperative complications. Patient-reported outcomes comprised the Oswestry Disability Index (ODI), lumbar pain (visual analog scale [VAS], 0–10; LVAS), radicular pain (VAS, 0–10; RVAS), patient-reported clinical improvement assessed at the postoperative consultation, and the self-reported percentage of improvement relative to the preoperative baseline, with 100% indicating complete recovery and 0% indicating no change.

### 2.5. Ethical Considerations

All procedures were conducted in accordance with institutional ethical standards and the Declaration of Helsinki. Informed consent for clinical data collection and analysis was obtained from all patients during their initial consultation. The study was approved by the institutional review board of the Société Française de Chirurgie rachidienne No. IRB00014665_SFCR_IRB #1_2026/10.

### 2.6. Statistical Analysis

Patient characteristics and preoperative and postoperative variables were compared between the SLD and MLD groups. Comparative analyses were conducted using Student’s t-test, the Mann–Whitney U test, or the Wilcoxon signed-rank test, depending on data normality and validity assumptions. All statistical analyses were performed using Jamovi (The Jamovi Project, version 2.5.6). The significance level was set at *p* < 0.05 for all tests.

## 3. Results

During the study period, 305 patients underwent surgery for lumbar spinal stenosis in our center. Among them, 83 patients (27%) presented with MLSS and were included in the present analysis. The population comprised 56.6% men (n = 47). The mean age at surgery was 74 years +/− SD 8.17 (range 56–92). Patients were retired in 87% of cases (n = 71). Patients were classified as ASA1 in 84% of cases (n = 56), ASA2 5% (n = 3) and ASA3 in 12% (n = 8).

Patients presented with lumbar pain in 83% of cases (n = 74) and radicular pain in 98% (n = 80), and 3 patients had motor deficit (4%). The mean preoperative ODI was 38.9 ± 15.4. Preoperatively, lumbar and radicular pain (VAS 0–10) both had a median of 7 [IQR 6–8].

All patients had a CT scan and MRI imaging. A radiculoscanner was necessary for decision-making in 18% of cases (n = 15). Within this subgroup, one patient (7%) presented with a mixed Schizas C/D grade stenosis, one patient (7%) had a double-level Schizas grade B stenosis, two patients (13%) presented with mixed Schizas B/C configurations and the predominant pattern was a double-level Schizas grade C stenosis, observed in the remaining patients (73.3%).

Patients had two compressed levels in 76% of cases (n = 63) and three levels in 24% (n = 20). Axial MRI slices suitable for grading were available for 71 of 83 patients. Among these, 45% (n = 32) had two levels graded Schizas C, 11% (n = 8) had two levels graded Schizas D, 38% (n = 27) had one level graded C and one level graded D, and 4% (n = 3) had one level graded B and one level graded C.

The operated level was classified as Schizas B in 3% (n = 2), Schizas C in 51% (n = 39), and Schizas D in 47% (n = 36). The non-operated levels were classified as Schizas B in 4% (n = 3), Schizas C in 85% (n = 61), and Schizas D in 11% (n = 8).

**Figure 5:**
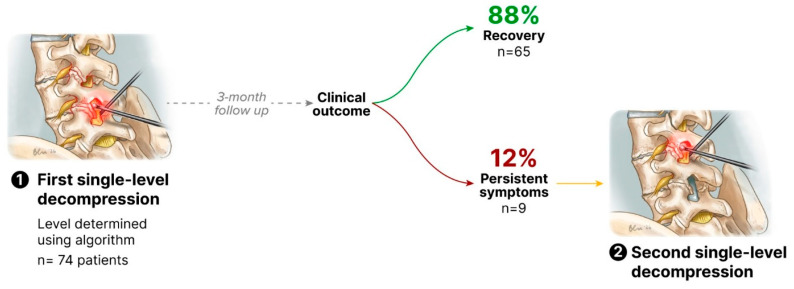
Schematic illustration of the surgical strategy and sequential management. Left: Initial procedure targeting the symptom-driving L4–L5 level using UBE. Right: Potential second procedure illustrating additional decompression at L3–L4 if clinically indicated; the L4–L5 level is already decompressed. The figure also illustrates the sequential management strategy consisting of initial SLD followed by clinical reassessment within 3 months. Among the 74 patients who underwent SLD, 9 required a second surgery during follow-up to address an additional compressed level. Copyright of Bridget Xiao-Cha Lu, used with permission.

The mean follow-up for the overall cohort was 238 ± 201 days. Among the 83 patients included in the study, 9 patients (11%) underwent an initial MLD, while the remaining 74 patients (89%) underwent an initial SLD. Among the patients treated with SLD, 9 (12%) required a secondary surgical procedure to decompress an additional level during follow-up. The remaining 65 patients (78% of the overall cohort and 88% of the SLD group) did not require further surgical intervention (Figure 5).

In the MLD group (n = 9), the most frequent decompression patterns were L2–L3 + L3–L4 and L3–L4 + L4–L5, each accounting for 44% (n = 4), whereas one patient (11%) underwent decompression at L2–L3 + L4–L5. In the SLD group (n = 74), the most commonly treated level was L4–L5 in 51% of cases (n = 38), followed by L3–L4 in 39% (n = 29) and L2–L3 in 7% (n = 5); L5–S1 and L1–L2 were rarely addressed, each in 1% of cases (n = 1). Among the nine SLD patients who subsequently underwent a second procedure, the additional decompression most frequently involved L3–L4 in 56% (n = 5), followed by L4–L5 in 33% (n = 3) and L2–L3 in 11% (n = 1).

A dural tear occurred in 1 patient (11%) in the MLD group and in 7 patients (8%) in the SLD group, with no significant difference between groups (*p* = 0.874). Postoperative complications consisted of one postoperative spondylodiscitis (1%) and one motor deficit with partial neurological recovery (1%). All patients were discharged after a one-day hospitalization.

The follow-up visit was conducted at 63.2 ± 11.2 days. At this visit, clinical improvement was documented in 91% of patients by the operating surgeon. At this point, patient-reported outcome questionnaires were available for 31 patients in the SLD group and 5 patients in the MLD group. The median paired difference for ODI was −13.2 points (*p* < 0.001; Wilcoxon-signed rank) and −3 points for both lumbar and radicular VAS (*p* < 0.01 respectively; Wilcoxon-signed rank).

Comparisons between the SLD and MLD groups are presented in Table 1. Patient self-reported percentage of clinical improvement averaged 67.1 ± 21.7% in the SLD group and 61.4 ± 20.5% in the MLD group, with no significant difference between groups (*p* = 0.58). No significant difference for ODI and VAS was reported between groups. The mean operative time was significantly longer in the MLD group (122 ± 28.1 min) compared with the SLD group (58.1 ± 12.0 min; *p* < 0.001).

Among the 9 patients who required a second surgical procedure in the SLD group, the mean interval between the two interventions was 248 ± 166 days (range: 84–532). Two patients (22%) underwent sequential decompression, with a Schizas grade D stenosis treated at the index surgery and a grade C stenosis addressed during the subsequent procedure. Four patients (44%) required repeat decompression of a Schizas grade C stenosis at two distinct levels, whereas three patients (33%) underwent repeat decompression of a Schizas grade D stenosis at two distinct levels.

Among the 8 patients presenting with Schizas grade D stenosis at both affected levels, three patients (38%) underwent initial MLD, whereas five patients (63%) were initially managed with SLD. Within the SLD subgroup, two patients (40%) did not require further surgery, while three patients (60%) subsequently required an unplanned second surgical intervention.

## 4. Discussion

The main finding of this study is that most patients with MLSS were safely and effectively managed with an SLD strategy, without need for further surgery after a mean follow up of 238 days. Of the 74 patients initially treated with SLD, only 12% (n = 9) required a secondary procedure to decompress an additional stenotic level during follow-up, while 87.8% (n = 65) achieved a favorable functional outcome without further surgical intervention. Taken together, these findings suggest that a stepwise, individualized surgical strategy for MLSS may represent a feasible approach in selected patients.

Almost one third of all patients operated on for lumbar spinal stenosis presented with MLSS, underscoring the clinical relevance of this condition. This is in line with reports of MLSS affecting from 35 to 40% of patients with lumbar spinal stenosis [4,12,13].

The challenge of determining the appropriate extent of decompression is not new. In the era of open spine surgery, several studies addressed this issue and reported less favorable outcomes following MLD compared with SLD [5,14,15]. Specifically, recovery in terms of ODI scores, pain intensity, and walking capacity was superior in patients undergoing SLD compared with those treated with MLD [5,14,15]. These results were largely attributed to the increased surgical invasiveness and paraspinal tissue damage associated with extensive open decompression. However, other studies have reported no significant differences in clinical outcomes between SLD and MLD [14,16,17,18].

With the advent of minimally invasive spine surgery, the question is even more relevant. For minimally invasive tubular decompression, Subramanian et al. reported similar outcomes for SLD or dual-level decompression [19]. With endoscopic spine surgery, Yoshikane et al. reported favorable clinical outcomes after SLD for MLSS using UBE [20]. Consistent with the literature, patients in our cohort showed improvement in their ODI scores as well as LVAS and RVAS postoperatively.

When choosing SLD, patients should be informed of the possible necessity of a second surgery; this was the case for 12% of our cohort. This reoperation rate is consistent with previously published series, with reported rates around 10% [20]. Importantly, severe stenosis has been shown to be associated with a substantially increased risk of reoperation compared with mild stenosis, with reported odds ratios approaching six [20]. Indeed, in our cohort, Schizas grade D stenosis at both affected levels emerged as a particularly challenging subgroup. Among these patients, most underwent initial MLD. When SLD was attempted, the likelihood of requiring a secondary procedure was high, with 60% of patients ultimately needing decompression of the second level. These findings suggest that double-level severe (Schizas grade D) stenosis may represent a threshold beyond which a two planned sequential SLDs could be considered, even within a minimally invasive framework. In this specific subgroup, larger studies comparing upfront MLD with planned staged sequential SLD strategies would be valuable to better define the optimal surgical approach.

In a recent retrospective study, Kim et al. reported no difference between SLD and MLD using UBE when it comes to back and leg VAS, complication and revision rates. However length of stay and time to ambulation were significantly longer in MLD [21]. These findings should be interpreted with caution and do not necessarily support a systematic MLD; instead, they suggest that a targeted SLD may be sufficient in carefully selected patients.

Surgical time was approximately twofold longer in the MLD group compared with the SLD group (122 ± 28.1 min vs. 58.1 ± 12.0 min), reflecting the increased procedural burden associated with multilevel endoscopic decompression. A targeted SLD strategy may be particularly relevant in the current phase of adoption of endoscopic spine surgery, which remains technically demanding and associated with a significant learning curve, often resulting in longer operative time and larger volume of irrigation saline [22]. In the setting of MLSS, extending decompression to additional levels, especially early in the learning curve, may increase operative complexity and duration without necessarily providing a clear clinical benefit in selected patients.

The safety profile of both approaches was favorable. Dural tears occurred in 8.4% of patients in the SLD group and in one patient in the MLD group. Importantly, none of these events required reoperation for persistent cerebrospinal fluid leakage or resulted in severe sequelae. Although the incidence of dural tears in our cohort was slightly higher than that reported by Yu et al., this discrepancy may be explained by the advanced age of our population, a well-recognized risk factor for dural fragility [23].

A radiculoscanner was required in 18% of cases to clarify symptom-level concordance, confirming that CT myelography remains a valuable adjunct in selected patients to refine surgical planning and limit unnecessary surgical extension. Although more invasive than MRI or standard CT, the radiculoscanner provides superior assessment of nerve root compression, particularly in the lateral recess and foraminal regions, and may help identify the single level most likely responsible for the patient’s symptoms. In our decision-making algorithm, CT myelography was used as a final step and was prescribed only after detailed patient information.

Finally, perioperative management outcomes further support the feasibility of UBE surgery in an elderly population. All patients were discharged after a one-day hospitalization, despite a mean age of 74 years. This rapid recovery likely reflects both the minimally invasive nature of UBE decompression and the implementation of enhanced recovery after surgery (ERAS) protocols, reinforcing the role of UBE as a particularly suitable technique for frail or elderly patients with lumbar spinal stenosis.

This study has several limitations. First, its retrospective single-center design may limit the generalizability of the findings and introduce inherent selection biases. Second, the mean follow-up duration was relatively short (238 ± 201 days), which may limit the ability to capture long-term outcomes, including delayed reoperations or late functional deterioration. Third, patient-reported outcome measures were available for only a subset of patients, with questionnaires completed by 36 patients. This limited sample size reduces the statistical power of patient-reported outcome comparisons and may introduce selection bias. However, clinical follow-up was available for all patients, and clinical improvement was systematically documented in the medical records at postoperative consultation. The limited availability of patient-reported outcome measures reflects the retrospective design of the study, in which standardized questionnaires were not consistently collected, rather than incomplete outcome assessment. Fourth, comparisons between the two groups were inherently unbalanced, as only 9 patients underwent MLD. This substantial discrepancy in group sizes limits the statistical power of between-group analyses. Finally, the MLD group consisted of patients treated prior to the implementation of the SLD strategy, potentially introducing temporal and learning-curve biases related to evolving surgical experience, perioperative management, and patient selection over time. Consequently, these findings should be interpreted cautiously and considered hypothesis-generating pending prospective validation.

## 5. Conclusions

Taken together, these findings suggest that a stepwise, individualized surgical strategy for MLSS may represent a feasible approach in selected patients. In our cohort, targeted SLD was associated with clinical improvement in most patients and shorter operative times. While these results support further investigation of SLD strategies within minimally invasive spine surgery, prospective controlled studies are needed before definitive conclusions can be drawn.

## Figures and Tables

**Table 1:** Baseline characteristics, perioperative variables, and clinical outcomes of patients undergoing SLD versus MLD for MLSS. Values are presented as n (%) or mean ± standard deviation. ODI: Oswestry Disability Index; ASA: American Society of Anesthesiologists physical status classification.

	SLD (n = 74)	MLD (n = 9)	*p*
Male	38 (51.4%)	9 (100%)	0.005
Mean age at surgery	74	73.9	0.973
ASA1/2/3	54/3/7	2/0/1	0.484
Lumbar pain present	66 (89.2%)	8 (88.9%)	0.662
Radicular pain present	71 (95.9%)	9 (100%)	0.615
Mean preoperative ODI	38.7	43	0.706
Radiculoscanner	15 (20.3%)	0	0.132
Dural tear	7 (9.5%)	1 (11.1%)	0.874
Complications	2 (2.7%)	0	0.618
Surgery duration	58.1 ± 12	122 ± 28.1	<0.0 01
Mean postoperative ODI	22.7	28.9	0.460

## Data Availability

The data that support the findings of this study are available from the corresponding author upon reasonable request.
